# Chronic Running Exercise Alleviates Early Progression of Nephropathy with Upregulation of Nitric Oxide Synthases and Suppression of Glycation in Zucker Diabetic Rats

**DOI:** 10.1371/journal.pone.0138037

**Published:** 2015-09-17

**Authors:** Daisuke Ito, Pengyu Cao, Takaaki Kakihana, Emiko Sato, Chihiro Suda, Yoshikazu Muroya, Yoshiko Ogawa, Gaizun Hu, Tadashi Ishii, Osamu Ito, Masahiro Kohzuki, Hideyasu Kiyomoto

**Affiliations:** 1 Department of Miyagi Community Health Promotion, Tohoku University Graduate School of Medicine, Tohoku University, Sendai, Japan; 2 Department of Internal Medicine and Rehabilitation Science, Tohoku University Graduate School of Medicine, Tohoku University, Sendai, Japan; 3 Department of Clinical Pharmacology and Therapeutics, Tohoku University Graduate School of Pharmaceutical Sciences and Medicine, Tohoku University, Sendai, Japan; 4 Department of Integrated Nephrology and Telemedicine, Tohoku Medical Megabank Organization, Tohoku University, Sendai, Japan; University of Louisville, UNITED STATES

## Abstract

Exercise training is known to exert multiple beneficial effects including renal protection in type 2 diabetes mellitus and obesity. However, the mechanisms regulating these actions remain unclear. The present study evaluated the effects of chronic running exercise on the early stage of diabetic nephropathy, focusing on nitric oxide synthase (NOS), oxidative stress and glycation in the kidneys of Zucker diabetic fatty (ZDF) rats. Male ZDF rats (6 weeks old) underwent forced treadmill exercise for 8 weeks (Ex-ZDF). Sedentary ZDF (Sed-ZDF) and Zucker lean (Sed-ZL) rats served as controls. Exercise attenuated hyperglycemia (plasma glucose; 242 ± 43 mg/dL in Sed-ZDF and 115 ± 5 mg/dL in Ex-ZDF) with increased insulin secretion (plasma insulin; 2.3 ± 0.7 and 5.3 ± 0.9 ng/mL), reduced albumin excretion (urine albumin; 492 ± 70 and 176 ± 11 mg/g creatinine) and normalized creatinine clearance (9.7 ± 1.4 and 4.5 ± 0.8 mL/min per body weight) in ZDF rats. Endothelial (e) and neuronal (n) NOS expression in kidneys of Sed-ZDF rats were lower compared with Sed-ZL rats (*p*<0.01), while both eNOS and nNOS expression were upregulated by exercise (*p*<0.01). Furthermore, exercise decreased NADPH oxidase activity, p47^phox^ expression (*p*<0.01) and α-oxoaldehydes (the precursors for advanced glycation end products) (*p*<0.01) in the kidneys of ZDF rats. Additionally, morphometric evidence indicated renal damage was reduced in response to exercise. These data suggest that upregulation of NOS expression, suppression of NADPH oxidase and α-oxoaldehydes in the kidneys may, at least in part, contribute to the renal protective effects of exercise in the early progression of diabetic nephropathy in ZDF rats. Moreover, this study supports the theory that chronic aerobic exercise could be recommended as an effective non-pharmacological therapy for renoprotection in the early stages of type 2 diabetes mellitus and obesity.

## Introduction

Sedentary behavior is known to increase obesity, aggravate type 2 diabetes mellitus (T2DM) and lead to early mortality [1]. Physical exercise training, especially regular aerobic exercise of moderate intensity, has numerous beneficial effects including reduction of body weight and improvement of insulin sensitivity, hyperglycemia, hyperlipidemia and diabetic nephropathy, which can prevent or attenuate the development of T2DM [1, 2]. However, the mechanisms underlying the beneficial action of exercise on diabetic nephropathy in T2DM are not yet fully understood.

Several studies have shown that exercise can be beneficial for diabetic nephropathy in obese Zucker rats [3, 4] and type 1 diabetic rats [5]. Furthermore, exercise can prevent insulin secretion failure through increased β-cell function and mass [6, 7], and decreased hepatic inflammation and oxidative stress [8] in Zucker diabetic fatty (ZDF) rats. These studies have largely focused on the pancreas and liver for potential mechanisms of exercise treatment in ZDF rats. However, the effects of exercise on kidney-related mechanisms in ZDF rats are unknown.

Nitric oxide (NO) is a vasodilator synthesized by three isoforms of NO synthase (NOS): endothelial (e), neuronal (n) and inducible (i) NOS. In the kidneys, NO has various protective effects, including regulation of renal hemodynamics, renin secretion, inhibition of tubular sodium reabsorption, tubuloglomerular feedback (TGF) and renal sympathetic nerve activity [9, 10]. Indeed, chronic NOS inhibition induced renal damage, proteinuria and glomerular sclerotic injury in rats [11], while eNOS and nNOS expression were downregulated in the kidneys of rats with chronic renal failure [12]. Furthermore, under diabetic conditions, nephropathy was aggravated using a NOS inhibitor (L-NAME) and reduced using an NO precursor (L-arginine) in Otsuka Long-Evans Tokushima Fatty rats [13]. The expression levels, localization and action of NOS are different in the various segments of the renal cortex and medulla [14, 15]. Renal vasa recta, glomeruli and afferent arterioles contain large amounts of constitutive NOS isoforms [14], while NO in the medulla contributes to improved blood flow [16]. Furthermore, nNOS expression in the macula densa of the cortex is considered a key mechanism for regulation of TGF and renin secretion [10, 17]. Recently, exercise was reported to produce antihypertensive and renoprotective effects with upregulation of renal eNOS and nNOS expression in spontaneously hypertensive rats (SHR) [18, 19]. We also showed that exercise restored cardiac and renal function with upregulation of eNOS and nNOS expression in the left ventricle, renal cortex and medulla in rats with chronic heart failure [20]. Thus, it is important to assess renal NOS expressions in both the renal cortex and medulla, to elucidate the mechanisms involved in the renal protective effects of exercise.

Oxidative stress contributes to progression of diabetic nephropathy [21, 22], and nicotinamide adenine dinucleotide phosphate (NADPH) oxidase is considered a major source of superoxide anion (O_2_^•–^) in diabetic nephropathy [21, 22]. NADPH oxidase is a multi-subunit enzyme that includes p47^phox^ [23], which is prominently expressed in podocytes [24]. Generated O_2_^•–^, in part, immediately reacts with NO to form the highly reactive intermediate peroxynitrite (ONOO^−^), which restricts NO bioavailability and leads to oxidative damage and cytotoxicity [23, 24].

Additionally, accumulation of advanced glycation end products (AGEs), which are produced by the modification of proteins from glucose with highly reactive carbonyl intermediates (α-oxoaldehydes) such as methylglyoxal (MG), 3-deoxy-D-glucosone (3DG) and glyoxal (GO), is an important risk factor for diabetic complications, including diabetic nephropathy [22].

Thus, the aim of the present study was to determine whether the beneficial effects of exercise on diabetic nephropathy in ZDF rats are related to renal oxidative stress; specifically levels of NADPH oxidase, glycation, especially of precursors of AGEs, and renal NOS.

## Materials and Methods

### Animals and ethics statement

Five-week-old male Zucker diabetic fatty (ZDF Lepr^fa/fa^; weighing 110–130 g, *n* = 10) rats and their control lean ZDF (^fa/+ or +/+^; 90–110 g, *n* = 5) counterparts were obtained from Charles River Laboratories Japan Inc. (Kanagawa, Japan). Rats were housed in cages (2 or 3 rats per cage) at the animal care facility of Tohoku University School of Medicine, with free access to water and standard laboratory chow (Labo MR Stock, Nosan Co., Tokyo, Japan) as previously described [19], in a temperature-controlled room (24°C) and under a 12-h light-dark cycle. All animal experiments were approved by the Tohoku University Committee for Animal Experiments (Approval No. 2013-Idou-583) and were performed in accordance with the Guidelines for Animal Experiments and Related Activities of Tohoku University, as well as the guiding principles of the Physiological Society of Japan and the US National Institutes of Health (NIH).

### Experimental groups and exercise training protocol

After 1 week of acclimatization, rats were randomized into sedentary (Sed-ZDF, *n* = 5) and exercise (Ex-ZDF, *n* = 5) groups. Lean ZDF rats were maintained as controls under sedentary conditions (Sed-ZL, *n* = 5). Rats in the Ex-ZDF group were allowed to run on a treadmill (KN-73; Natsume Industries Co., Tokyo, Japan) for 10 min/day at an initial treadmill speed of 10 m/min on a 0% gradient. Treadmill speed was increased gradually over 1 week to 20 m/min and the duration of exercise was increased to 60 min/day. This graduated protocol is appropriate for rats as a chronic aerobic exercise model, as we and other authors have previously reported.[4, 19, 20, 25]. The peak oxygen consumption (VO_2_) was measured after 1 week of acclimatization for exercise by an oxygen-carbon dioxide metabolism measuring system within a sealed chamber treadmill (Model MK-5000; Muromachikikai, Tokyo, Japan), as described previously [20]. From these studies, 20 m/min was determined as an aerobic level for the rats. The VO_2_ when rats were running at 20 m/min corresponded to approximately 50–70% of the peak VO_2_. Treadmill exercise (10–60 min/day, 5 days/week) was performed for 8 weeks (from 6 to 14 weeks of age).

### Water and food intake, body weight, plasma and urinary parameters and blood pressure

The water and food intake and body weight were measured weekly. After the exercise protocol, all rats were housed in individual metabolic cages (Model ST; Sugiyama-General, Tokyo, Japan) for 3 days to acclimatize to the conditions. Urine samples were collected on ice over 24 h. Urinary albumin concentrations were determined using a commercially available assay kits (AKRAL-120; Shibayagi, Co., Gunma, Japan), as described previously [20]. Systolic blood pressure (SBP) was measured after urine collection from conscious rats using an indirect tail-cuff method (Model UR-5000; Ueda, Tokyo, Japan), as described previously [20]. Five days after the last exercise session and 30–60 min of fasting, all rats were anesthetized with sodium pentobarbitone (50 mg/kg, i.p.) and then blood samples were collected by decapitation. Blood samples were centrifuged for 5 min at 1500 ×*g* and the supernatant collected and stored at −80°C. Plasma glucose, total cholesterol, triglyceride, free fatty acid, blood urea nitrogen, creatinine, urinary creatinine and uric acid were determined by standard auto-analysis techniques (BML, Tokyo, Japan).

### Glucose tolerance test

Four days after the last exercise session and at midday after 5 h of fasting, all rats were subjected to an intra-peritoneal glucose tolerance test (IPGTT) (2 g glucose/kg body weight), as previously described with some modifications [6, 8]. Tail blood was collected at 30-min intervals starting at time 0, just before injection, and continuing for 120 min. Glucose concentrations were measured with a blood glucose meter (Medisafemini GR-102; Terumo Co., Tokyo, Japan). Plasma insulin was measured with commercial kits (Rat Insulin ELISA Kit; Shibayagi). Homeostasis model assessment for insulin resistance (HOMA-IR), HOMA for insulin sensitivity (HOMA-IS) and HOMA for β-cell function (HOMA-β) were calculated, as previously described [26], according to the following equations:

$$HOMA-IR=\frac{\text{fasting insulin (ng/mL)}\times \text{fasting glucose (mg/dL)}}{405}.$$

$$HOMA-IS=\frac{10000}{\text{fasting insulin (ng/mL)}\times \text{fasting glucose (mg/dL)}}$$

$$HOMA-\beta =\frac{\text{fasting insulin (ng/mL)}\times 20}{\text{fasting glucose (mg/dL)}-3.5}$$

### Measurement of renal nitric oxide synthase activity

Five days after the last exercise session, after all rats had been decapitated, their kidneys were quickly removed. One kidney per rat was hemisected and dissected into the cortex and outer medulla. The other kidney was fixed in 10% formalin for histological analysis. Dissected tissues were homogenized in 100 mmol/L potassium buffer (pH 7.25) containing 30% glycerol, 1 mmol/L dithiothreitol and 0.1 mmol/L phenylmethylsulfonyl fluoride. The samples were snap-frozen in liquid nitrogen and stored at −80°C. Protein concentrations in the samples were determined by the Bradford method [27] with bovine γ-globulin as a standard (Bio-Rad Laboratories, Hercules, CA, USA).

For determination of NOS activity, the *in vitro* formation of nitrate/nitrite (NO_x_) by each tissue was evaluated using a commercially available kit (Oxford Biomedical Research, Rochester Hills, MI, USA), as described previously [19].

### Western blot analysis

Protein expression of nitric oxide synthase, nitrotyrosine as an index of ONOO^−^ formation and p47^phox^ were examined by western blot analysis, as described previously [15, 19]. Briefly, protein samples (50 μg) were separated by electrophoresis on a sodium dodecyl sulfate polyacrylamide gel (5.8, 10 and 12%, as appropriate for each molecular weight) and blotted onto nitrocellulose membrane. The membrane was incubated for 2 h at room temperature with primary antibodies raised against eNOS and nNOS (diluted 1:2000 and 1:500, respectively; BD Transduction Laboratories, San Diego, CA, USA), nitrotyrosine and p47^phox^ (1:1000; Santa Cruz Biotechnology, Dallas, TX, USA). It was then incubated with horseradish peroxidase-conjugated anti-mouse and anti-rabbit IgG secondary antibodies, as appropriate for each primary antibody (1:2000; Santa Cruz Biotechnology) for 1 h at room temperature. The immunoblots were developed with an enhanced chemiluminescence kit (Super Signal; Thermo Fisher Scientific, Waltham, MA, USA). The relative intensities of bands for each protein were quantified by ImageJ (v1.48; NIH, Bethesda, MD, USA). The intensities of the bands for each protein were normalized against those for *β*-actin as an internal standard. The intensity of the bands in the Sed-ZL rats was assigned a value of 1.

### Level of thiobarbituric acid-reactive substances (TBARS)

Plasma and urinary thiobarbituric acid-reactive substances (TBARS) were measured as an index of lipid peroxidation using a colorimetric assay kit (Cayman Chemical, Ann Arbor, MI, USA), as described previously [19]. Data are expressed as nmol malondialdehyde (MDA)/mL for plasma TBARS and nmol MDA/day for urinary TBARS.

### NADPH oxidase activity

NADPH oxidase activity was measured as an index of O_2_^•–^ generation by a lucigenin-enhanced chemiluminescence method, as described previously [19]. Briefly, proteins from renal cortical samples (200 μg) were resuspended in 1 ml Krebs’-HEPES buffer (composition (in mmol/L): NaCl 119; HEPES 20; KCl 4.6; CaCl_2_ 1.2; Na_2_HPO_4_ 0.15; KH_4_PO_4_ 0.4; MgSO_4_ 1.0; NaHCO_3_ 25; glucose 5.5). Background chemiluminescence was recorded using a tube luminometer (PSN AB-2200; ATTO, Tokyo, Japan) for 5 min after the addition of lucigenin (10 μmol/L; Sigma-Aldrich, St Louis, MO, USA). After the addition of NADPH (100 μmol/L), chemiluminescence was measured for an additional 5 min. The activity of NADPH oxidase was determined by subtracting background values from values obtained after the addition of NADPH and expressed as c.p.m./g protein.

### Quantification of α-oxoaldehydes (MG, 3DG and GO)

α-oxoaldehydes were assayed by derivatization with o-phenylenediamine (o-PD) (Wako Pure Chemical Industries, Osaka, Japan) and triple quadrupole mass spectrometry (MS) of the resulting quinoxalines, as previously described with some modifications [28]. Perchloric acid (40 μL, 5 M) was added to 200 μL of urine or tissue homogenate solution. Samples were incubated on ice for 10 min and centrifuged (18,000 ×*g*, 10 min) to remove the precipitate. A 50- μL aliquot of 10 μM 2, 3-butanedione (Wako Pure Chemical Industries) as internal standard and 50 μL of 100 mM of o-PD were added to 100 μL of the supernatant, and then incubated at 4°C for 22 h. Samples (100 μL) were then loaded into the prepared Sep-Pak tC18 μElution plate (Waters Co., Milford, MA, USA), rinsed with 200 μL of 0.05% formic acid and eluted twice with 25 μL 0.05% formic acid. Eluted samples were then filtrated through 0.2 μm filters (Merck Ltd, Tokyo, Japan) into sample vials.

**Fig 1:**
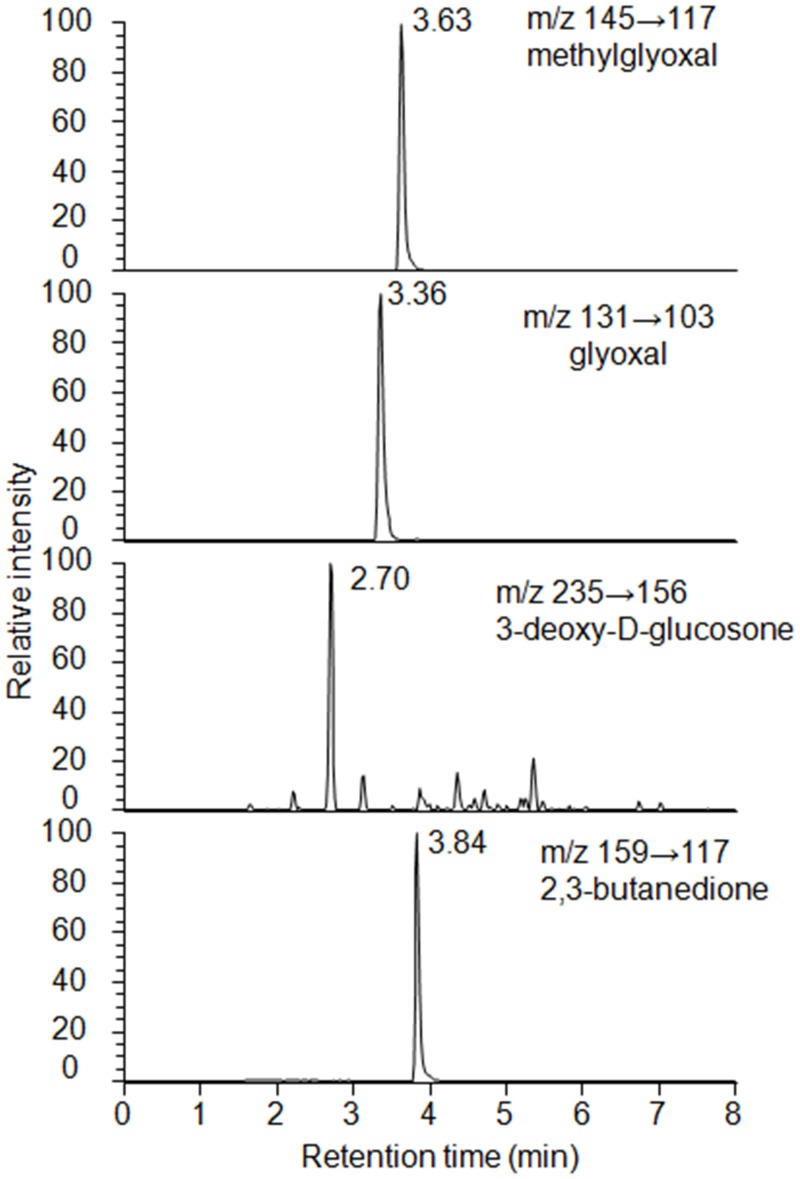
Chromatograms of derived methylglyoxal, glyoxal, 3-deoxy-D-glucosone and 2,3-butanedione. The concentration of each substance tested was 6.25 pmol. The ion transition of the derived methylglyoxal, glyoxal, 3-deoxy-D-glucosone and 2,3-butanedione was *m/z* 145→117, *m/z* 131→103, *m/z* 235→156, and *m/z* 159→117, respectively.

Quantitative analysis of the derivatized α-oxoaldehydes by electrospray ionization (ESI) liquid chromatography (LC)-MS/MS was performed with a Nexera Quanternary LC Systems (Shimadzu Co., Tokyo, Japan) coupled to a TSQ Quantum Ultra (Thermo Fisher Scientific) and operated in the positive ion mode. Five microliters of each sample was injected onto a 75- × 2.0-mm Shim-pack XR ODS III (1.6 μm) (Shimadzu) with a flow rate of 0.4 mL/min. For gradient elution, mobile phase A was H_2_O/acetonitrile/HCOOH = 98/2/0.05 and mobile phase B was H_2_O/acetonitrile/HCOOH = 2/98/0.05. The linear and stepwise gradient was programmed as follows; 0–1 min: 10–20% solvent B; 1–3 min: 20–60% solvent B; 3–4 min: 60–100% solvent B; 4–6 min: 100% solvent; 6.1–8 min: 10% solvent B. Derivatized MG, 3DG, GO and 2,3-butanedione were detected in selected reaction monitoring mode, monitoring the transitions of *m/z* 145–117, *m/z* 235–156, *m/z* 131–103 and *m/z* 159–117, respectively (Fig 1). Positive ion mode monitoring was selected. Spray voltage was 3000 V, vaporizer temperature was 350°C and ion transfer tube temperature was 300°C. Values of renal tissues are expressed as the amount of the α-oxoaldehydes per gram of the tissue protein.

### Renal histology

#### Histopathological analysis of renal injury

The kidneys were fixed in 10% formalin. Paraffin sections (3 μm) were prepared and stained with Periodic acid-Schiff (PAS) to evaluate the degree of glomerular injury. At least 100 glomeruli per rat were scored as an index of glomerular sclerosis (IGS) in a blinded fashion on a 0–4 scale; 0 representing a normal glomerulus, 1 representing loss of 1–25% of glomerular capillary area, 2 representing a 26–50% loss, 3 representing a 51–75% loss and 4 representing >75% loss of the capillaries in the glomerular tuft. Images were captured using a Nikon Eclipse 80i microscope equipped with a Nikon DS-Fi2-U3 color camera (Nikon Instruments Inc., Tokyo, Japan).

### Immunohistochemistry

The kidney tissues were fixed with 10% paraformaldehyde and embedded in paraffin. Tissue sections (3 μm) were deparaffinized in xylene, rehydrated in graded ethanol, and then rinsed in the phosphate-buffered saline (PBS). To block endogenous peroxidase activity, rehydrated sections were treated with 0.3% H_2_O_2_ in absolute ethanol for 5 min, then processed for immunostaining with antibodies against anti-human alpha-smooth muscle actin (α-SMA) and desmin (diluted 1:300 and 1:150, respectively; Dako, Glostrup, Denmark), and a Histofine Simple Stain Max PO kits (Nichirei, Tokyo, Japan) according to the manufacturer’s instructions. After washing with PBS, a chromogen solution (diaminobenzidine and H_2_O_2_) was applied to the sections. The slides were counterstained with hematoxylin for 30 s. For each specimen, at least 30 randomly chosen cortical fields were photographed using a digital color camera (200× or 400× total magnification) and were quantified using ImageJ software (v1.48, NIH). We set color split channels with blue and adjusted threshold with 0–130, then the percentage of the target stained area was measured after the selection of a glomerular area for desmin. Two researchers performed all morphometric analyses of the kidney samples in a blinded manner.

### Statistical analysis

Comparisons between the groups were performed using one-way ANOVA followed by Tukey’s *post hoc* test or Kruskal–Wallis test with Exact Tests, as appropriate. Food intake, water intake, body weight and IPGTT data were assessed by two-way repeated-measures ANOVA with group × time interaction. If the ANOVA showed a significant effect, further *post hoc* analysis was performed by Scheffe tests. All statistical tests were performed with IBM SPSS Statistics, Version 22.0 (IBM Corp., Armonk, NY, USA). Two-tailed *p*-values of *p* < 0.05 were considered statistically significant. Data are presented as means ± SEM.

## Results

### Plasma and urine parameters, urine volume, and systolic blood pressure

Table 1 summarizes the plasma and urine parameters and hemodynamic data of the three groups at the end of the experiment. Plasma levels of glucose, total cholesterol, triglycerides, free fatty acids and blood urea nitrogen were significantly higher in the Sed-ZDF group than those in the Sed-ZL group (all *p*<0.01), and plasma creatinine level was significantly lower in the Sed-ZDF than the Sed-ZL group (*p*<0.01). Plasma levels of glucose and total cholesterol were also significantly lower in the Ex-ZDF group than the Sed-ZDF group (both *p*<0.01).

**Table 1:** Effects of chronic running exercise on plasma parameters, urine volume, urine parameters, and systolic blood pressure in Zucker diabetic fatty rats.

	Sed-ZL	Sed-ZDF	Ex-ZDF
**Plasma**			
Glucose (mg/dL)	168 ± 18	458 ± 12**	140 ± 7^††^
Total cholesterol (mg/dL)	76 ± 3	157 ± 6**	123 ± 6** ^††^
Triglycerides (mg/dL)	47 ± 6	190 ± 16**	165 ± 24**
Free fatty acids (mEq/L)	0.15 ± 0.01	0.29 ± 0.03**	0.24 ± 0.01*
Blood urea nitrogen (mg/dL)	18.8 ± 1.0	26.3 ± 1.6**	23.9 ± 1.0*
Creatinine (mg/dL)	0.29 ± 0.02	0.19 ± 0.01**	0.20 ± 0.01**
**Urine**			
Volume (mL/day)	7.3 ± 1.3	37.1 ± 7.4*	5.5 ± 0.7^†^
Uric acid (mg/day)	1.5 ± 0.2	1.7 ± 0.7	0.9 ± 0.1
Albumin:creatinine ratio (mg/g Creatinine)	50.4 ± 6.7	492.1 ± 69.9**	176.4 ± 10.5^††^
Creatinine clearance/body weight (mL/min per 100 g)	5.5 ± 0.7	9.7 ± 1.4*	4.5 ± 0.8^††^
**Hemodynamic data**			
Systolic blood pressure (mmHg)	148 ± 6	111 ± 3**	112 ± 3**

Sed-ZL, sedentary Zucker lean rats; Sed-ZDF, sedentary Zucker diabetic fatty rats; Ex-ZDF, treadmill exercised Zucker diabetic fatty rats.

* *p* < 0.05,

** *p* < 0.01 compared with Sed-ZL group.

^†^ *p* < 0.05,

^††^ *p* < 0.01 compared with Sed-ZDF group.

Values are mean ± SEM (*n* = 5/group).

Volume of urine per day was approximately fivefold higher in the Sed-ZDF group than the Sed-ZL group (*p*<0.05), whereas there was no significant difference between the Sed-ZL and Ex-ZDF groups. Although there was no significant difference in uric acid between the groups, the albumin-to-creatinine ratio and the creatinine clearance-to-bodyweight ratio were significantly higher in the Sed-ZDF group compared with the Sed-ZL group (approximately tenfold; *p*<0.01 and twofold; *p*<0.05, respectively), whereas the ratios were significantly lower in the Ex-ZDF group compared with the Sed-ZDF group (both *p*<0.01).

SBP was significantly lower in both the Sed-ZDF and Ex-ZDF groups compared with the Sed-ZL group (*p*<0.01).

### Weekly water and food intake, and body weight

**Fig 2:**
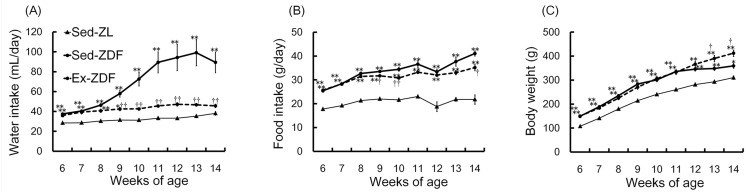
Effects of chronic running exercise on water intake, food intake and body weight over 8 weeks in Zucker diabetic fatty rats. (A) The water intake, (B) food intake and (C) body weight were measured weekly for 8 weeks in sedentary lean Zucker diabetic fatty (ZDF) rats (Sed-ZL), sedentary ZDF rats (Sed-ZDF) and aerobic treadmill exercised ZDF rats (Ex-ZDF) groups. Values are means ± SEM (*n* = 5/group). * *p*<0.05, ** *p*<0.01 compared with the Sed-ZL group; ^†^ *p*<0.05, ^††^ *p*<0.01 compared with the Sed-ZDF group.

Animal status was observed over 8 weeks as shown in Fig 2. Rats of both ZDF groups consumed significantly higher amounts of water (Fig 2A) and food (Fig 2B) compared with the Sed-ZL group at the start of the study (all *p*<0.01). Volume of water intake in the Sed-ZDF group gradually increased over the first 7 weeks, and was nearly twofold higher compared with the Sed-ZL and Ex-ZDF groups at 14 weeks of age (both *p*<0.01). During the 8 weeks of the study, food intake was significantly higher in both ZDF groups compared with the Sed-ZL group (both *p*<0.01). At 14 weeks of age, food intake was significantly higher in the Sed-ZDF group compared with the Sed-ZL (approximately twofold; *p*<0.01) and Ex-ZDF (approximately 20%; *p*<0.05) groups. Body weight (Fig 2C) was significantly higher in both ZDF groups compared with the Sed-ZL group (both *p*<0.01) at the start of the study. However, at 14 weeks of age, body weight was significantly higher in the Ex-ZDF group (412 ± 13 g) compared with the Sed-ZL (310 ± 7 g; *p*<0.01) and Sed-ZDF (359 ± 9 g; *p*<0.05) groups.

### IPGTT plasma glucose and insulin levels, and homeostasis model assessment

**Fig 3:**
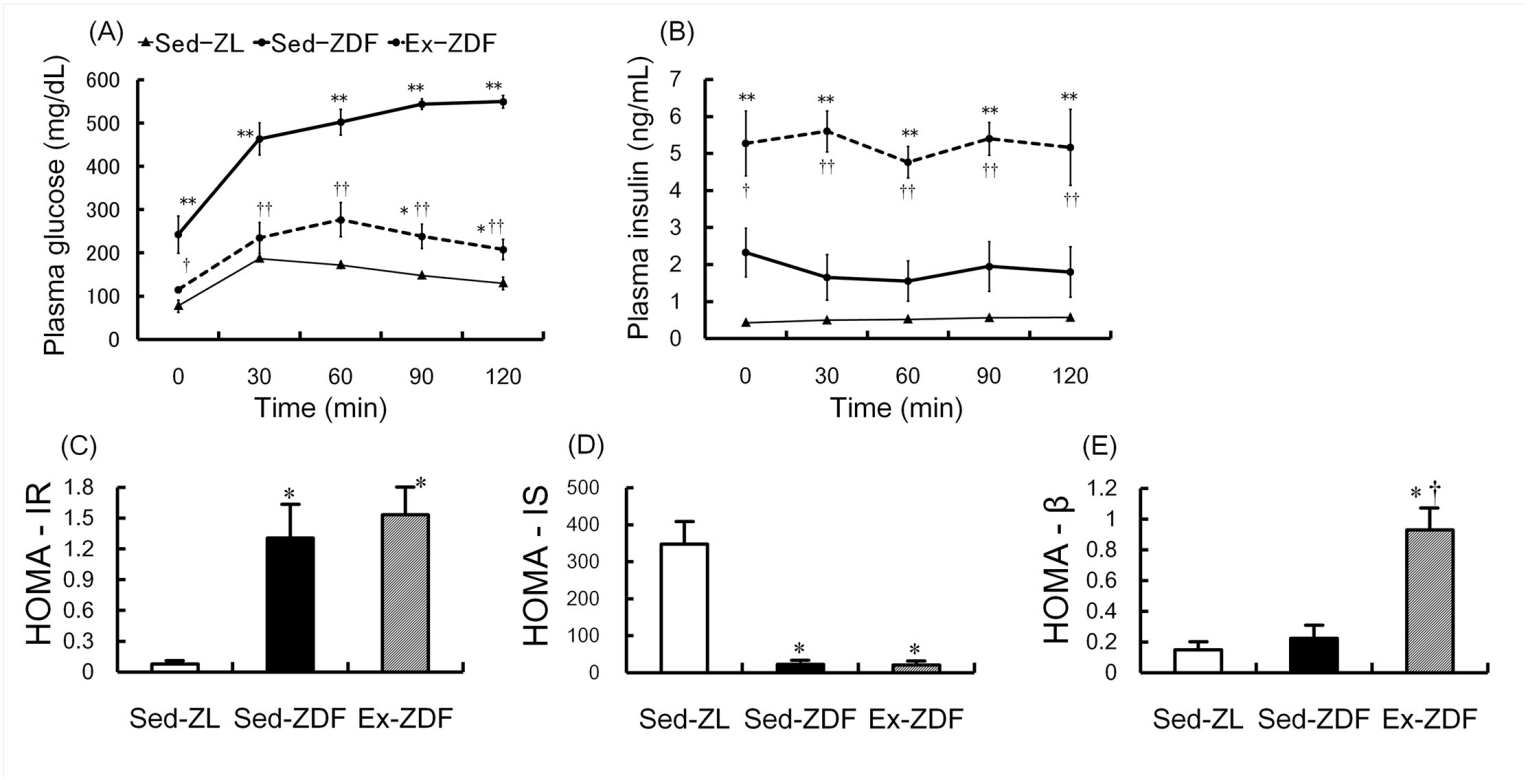
Effects of chronic running exercise on the intra-peritoneal glucose tolerance test in Zucker diabetic fatty rats. (A) Plasma glucose and (B) plasma insulin levels were measured by intra-peritoneal glucose tolerance test (IPGTT) at 4 days after the last exercise session and after 5 h of fasting in sedentary lean Zucker diabetic fatty (ZDF) rats (Sed-ZL), sedentary ZDF rats (Sed-ZDF) and aerobic treadmill exercised ZDF rats (Ex-ZDF) groups. (C) Homeostasis model assessment (HOMA) for insulin resistance (HOMA-IR), (D) HOMA for insulin sensitivity (HOMA-IS) and (E) HOMA for β-cell function (HOMA-β) were calculated by IPGTT data in Sed-ZL, Sed-ZDF and Ex-ZDF groups. Values are means ± SEM (*n* = 5/group). * *p*<0.05, ** *p*<0.01 compared with the Sed-ZL group; ^†^ *p*<0.05, ^††^ *p*<0.01 compared with the Sed-ZDF group.

The deterioration of plasma glucose and insulin levels with time over 8 weeks of intervention is shown in Fig 3A and 3B. As expected, glucose levels (Fig 3A) in the Sed-ZDF group (242 ± 43 mg/dL) were significantly higher compared with the Sed-ZL (77 ± 14 mg/dL; *p*<0.01) and Ex-ZDF groups (115 ± 5 mg/dL; *p*<0.05) before glucose injection. Glucose levels in the Sed-ZDF group continued to increase until 2 h after glucose injection, in contrast to the Sed-ZL and Ex-ZDF groups in which glucose levels decreased. At the last time point, glucose levels were significantly higher in the Sed-ZDF group (550 ± 14 mg/dL) compared with Sed-ZL (129 ± 14 mg/dL, approximately 4.2-fold) and Ex-ZDF (208 ± 24 mg/dL, approximately 2.6-fold) groups (both *p*<0.01). There was no significant interaction of group × time in levels of plasma insulin (Fig 3B). However, an independent insulin concentration test showed that plasma insulin levels were significantly different among the groups at all time points. At baseline, the insulin levels in the Ex-ZDF group (5.28 ± 0.88 ng/mL) were significantly higher compared with the Sed-ZL (0.43 ± 0.02 ng/mL; *p*<0.01) and Sed-ZDF (2.33 ± 0.66 ng/mL; *p*<0.05) groups.

HOMA-IR and HOMA-IS (Fig 3C and 3D), as indices of insulin resistance and sensitivity respectively, showed significantly higher insulin resistance and lower sensitivity in both ZDF groups compared with the Sed-ZL group (all *p*<0.05). By contrast, HOMA-β (Fig 3E), as an index for β-cell function, was significantly higher in the Ex-ZDF group compared with the Sed-ZL and Sed-ZDF groups (both *p*<0.05).

### Nitric oxide synthase activity and protein expression

**Fig 4:**
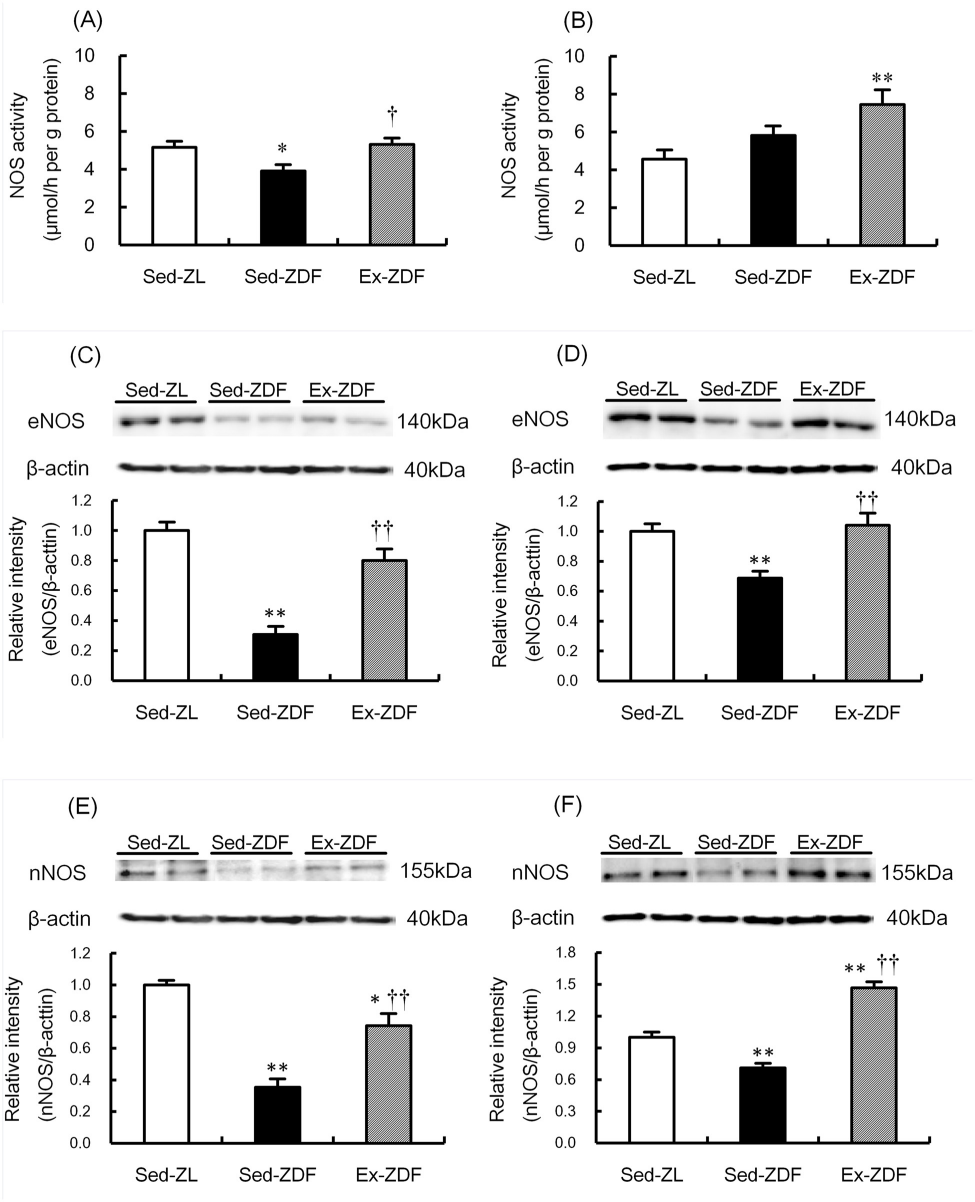
Effects of chronic running exercise on nitric oxide synthase (NOS) activity, endothelial NOS (eNOS) and neuronal NOS (nNOS) protein expression in the renal cortex and outer medulla of Zucker diabetic fatty rats. A, B: Nitric oxide synthase (NOS) activity was evaluated as the *in vitro* formation of nitrate/nitrite (NO_x_) in the renal cortex (A) and outer medulla (B) of sedentary lean Zucker diabetic fatty (ZDF) rats (Sed-ZL), sedentary ZDF rats (Sed-ZDF) and aerobic treadmill exercised ZDF rats (Ex-ZDF) groups. C–F: Protein expression of the endothelial NOS (eNOS) in the renal cortex (C) and outer medulla (D), and the neuronal NOS (nNOS) in the renal cortex (E) and outer medulla (F) of Sed-ZL, Sed-ZDF and Ex-ZDF groups were examined by western blot analysis. Top panels depict representative immunoblots from the different groups. The intensities of the eNOS bands (140 kDa) and the nNOS bands (155 kDa) for each protein were normalized against that of *β*-actin (40 kDa). The intensity of the band in the Sed-ZL group was assigned a value of 1. Values are means ± SEM (*n* = 5/group). * *p*<0.05, ** *p*<0.01 compared with the Sed-ZL group; ^†^ *p*<0.05, ^††^ *p*<0.01 compared with the Sed-ZDF group.

NOS activity and protein expression of the three groups are shown in Fig 4. NOS activity was significantly lower (by 24%; *p*<0.05) in the renal cortex of the Sed-ZDF group compared with the Sed-ZL group, and was significantly higher (by 36%; *p*<0.05) in the Ex-ZDF group compared with the Sed-ZDF group (Fig 4A). In the renal outer medulla (Fig 4B), NOS activity was significantly higher (by 63%) in the Ex-ZDF group compared with the Sed-ZL group (*p*<0.01).

eNOS protein levels were significantly lower in the renal cortex (Fig 4C) and outer medulla (Fig 4D) of the Sed-ZDF group compared with the Sed-ZL group (by 69%; *p*<0.01 and 31%; *p*<0.01, respectively). However they were significantly higher in the renal cortex and outer medulla of the Ex-ZDF group compared with the Sed-ZDF group (by 159%; *p*<0.01 and 51%; *p*<0.01, respectively). nNOS protein levels were also significantly lower in the renal cortex (Fig 4E) and outer medulla (Fig 4F) of the Sed-ZDF group compared with the Sed-ZL group (by 65%; *p*<0.01 and 29%; *p*<0.01, respectively). In contrast, nNOS protein levels were significantly higher in the renal cortex and outer medulla of the Ex-ZDF group compared with the Sed-ZDF group (by 110%; *p*<0.01 and 106%; *p*<0.01, respectively).

### Levels of thiobarbituric acid-reactive substances (TBARS)

**Fig 5:**
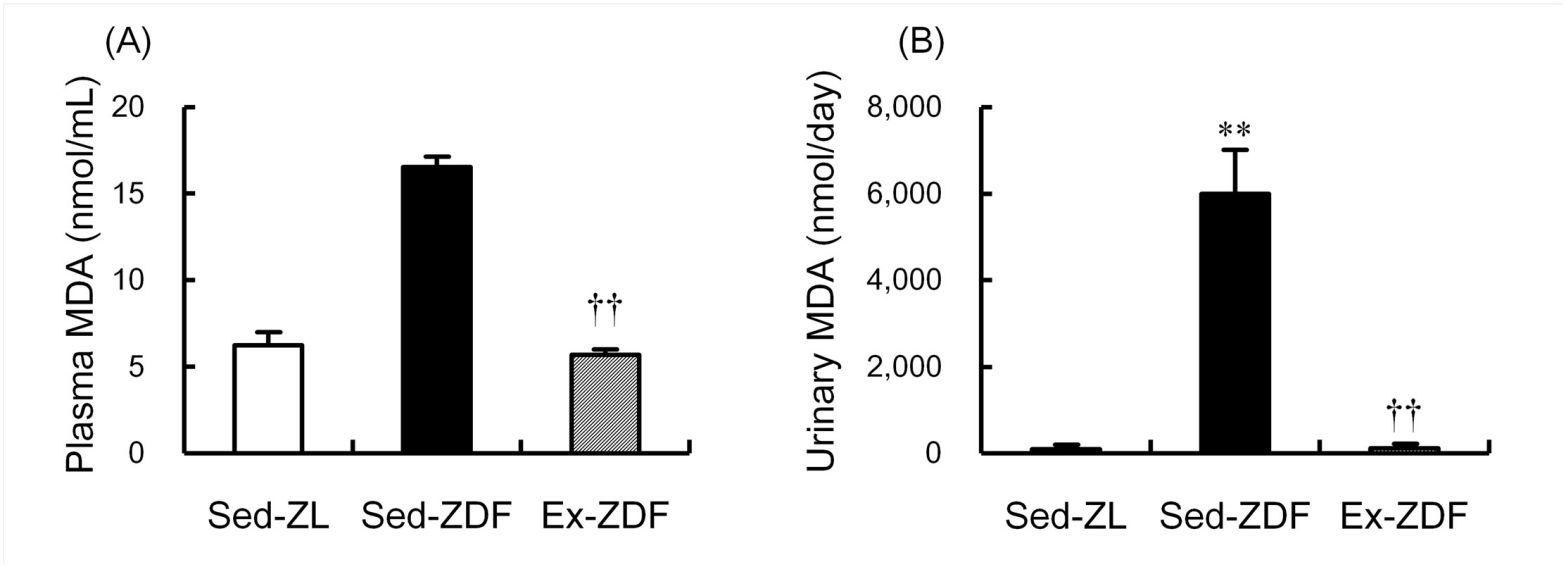
Effects of chronic running exercise on levels of thiobarbituric acid-reactive substances levels in Zucker diabetic fatty rats. (A) Plasma and (B) urinary thiobarbituric acid-reactive substances (TBARS) levels, expressed as malondialdehyde (MDA) concentrations, were measured as an index of lipid peroxidation in sedentary lean Zucker diabetic fatty (ZDF) rats (Sed-ZL), sedentary ZDF rats (Sed-ZDF) and aerobic treadmill exercised ZDF rats (Ex-ZDF) groups. Values are means ± SEM (*n* = 5/group). ** *p*<0.01 compared with the Sed-ZL group; ^††^ *p*<0.01 compared with the Sed-ZDF group.

Although there was no significant difference in plasma TBARS levels between the Sed-ZL and Sed-ZDF groups, exercise significantly decreased plasma TBARS in ZDF rats by 66% (*p*<0.01, Fig 5A). Urinary TBARS levels were significantly higher (nearly 65-fold; *p*<0.01) in the Sed-ZDF group compared with the Sed-ZL group, while exercise significantly decreased (nearly 50-fold; *p*<0.01) urinary TBARS in ZDF rats (Fig 5B).

### NADPH oxidase activity and p47^phox^ protein expression

**Fig 6:**
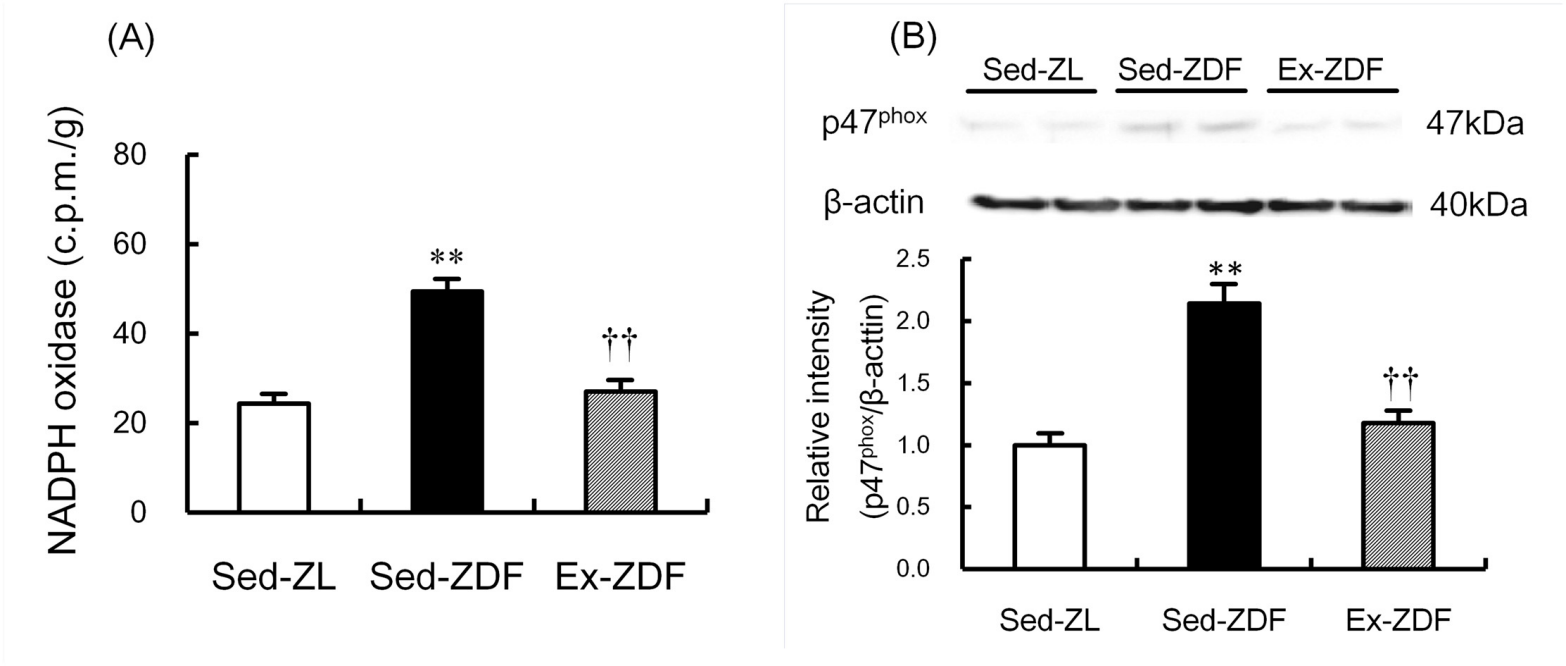
Effects of chronic running exercise on NADPH oxidase activity and p47^phox^ protein expression in the renal cortex of Zucker diabetic fatty rats. (A) NADPH oxidase activity was measured as an index of O_2_^•–^ generation in the renal cortex of sedentary lean Zucker diabetic fatty (ZDF) rats (Sed-ZL), sedentary ZDF rats (Sed-ZDF) and aerobic treadmill exercised ZDF rats (Ex-ZDF) groups. (B) Protein expression of the p47^phox^ in the renal cortex of Sed-ZL, Sed-ZDF and Ex-ZDF groups were examined by western blot analysis. Top panels depict representative immunoblots from the different groups. The intensities of the p47^phox^ bands (47 kDa) for the protein were normalized against *β*-actin (40 kDa). The intensity of the band in the Sed-ZL group was assigned a value of 1. Values are means ± SEM (*n* = 5/group). ** *p*<0.01 compared with the Sed-ZL group; ^††^ *p*<0.01 compared with the Sed-ZDF group.

NADPH oxidase activity (Fig 6A) was significantly higher in the renal cortex of Sed-ZDF group compared with the Sed-ZL group (by 99%; *p*<0.01), and there was no significant difference between the Ex-ZDF and Sed-ZL groups. Expression of p47^phox^ protein (Fig 6B) was significantly higher in the renal cortex of the Sed-ZDF group compared with the Sed-ZL group (by 214%; *p*<0.01), and there was no significant difference between the Ex-ZDF and Sed-ZL groups.

### Nitrotyrosine protein expression

**Fig 7:**
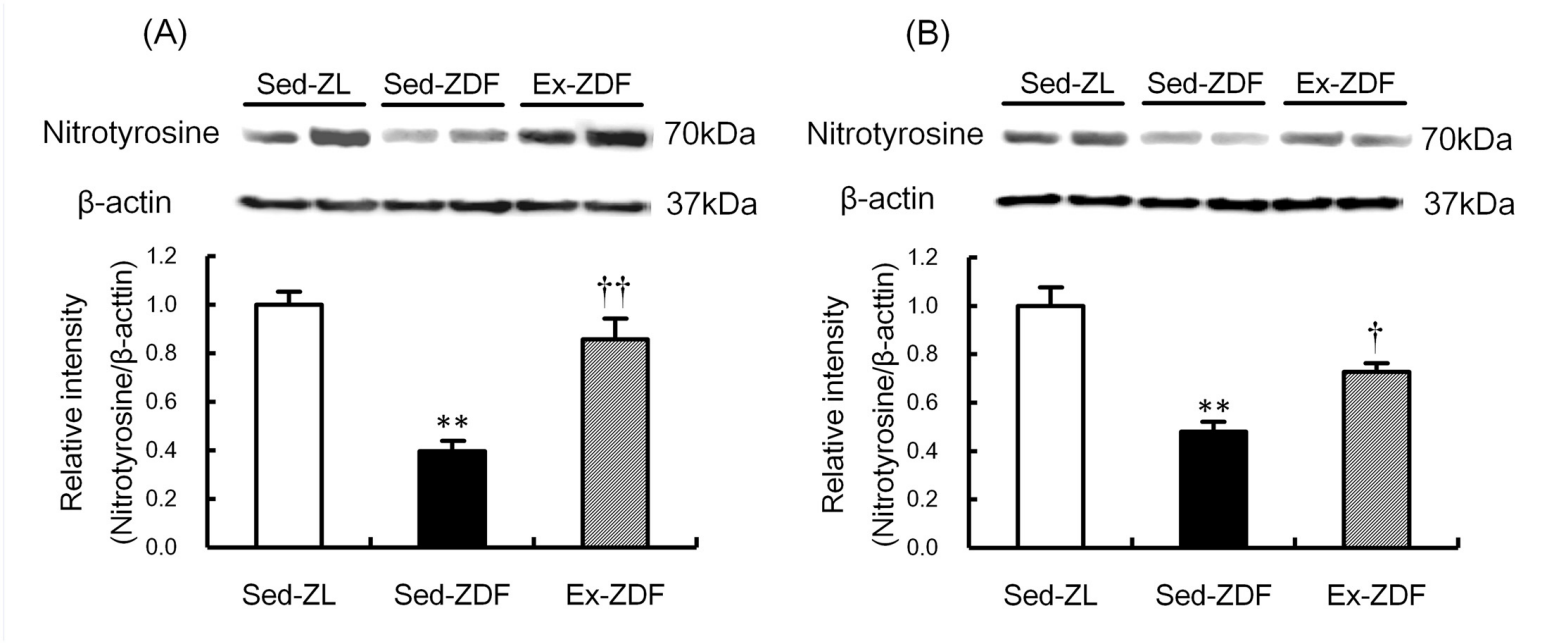
Effects of chronic running exercise on nitrotyrosine protein expression in the renal cortex and outer medulla of Zucker diabetic fatty rats. Protein expression of the nitrotyrosine in the renal cortex (A) and outer medulla (B) of sedentary lean Zucker diabetic fatty (ZDF) rats (Sed-ZL), sedentary ZDF rats (Sed-ZDF) and aerobic treadmill exercised ZDF rats (Ex-ZDF) groups were examined by western blot analysis. Top panels depict representative immunoblots from the different groups. The intensities of the nitrotyrosine bands (70 kDa) for the protein were normalized against that of *β*-actin (40 kDa). The intensity of the band in the Sed-ZL group was assigned a value of 1. Values are means ± SEM (*n* = 5/group). ** *p*<0.01 compared with the Sed-ZL group; ^†^ *p*<0.05, ^††^ *p*<0.01 compared with the Sed-ZDF group.

Nitrotyrosine protein levels in the three groups are shown in Fig 7. Nitrotyrosine protein expression was significantly lower in the renal cortex (Fig 7A) and outer medulla (Fig 7B) of the Sed-ZDF group compared with the Sed-ZL group (by 60%; *p*<0.01 and 52%; *p*<0.01, respectively), and no significant difference was observed in the kidney between the Ex-ZDF and Sed-ZL groups.

### Production of α-oxoaldehydes

**Fig 8:**
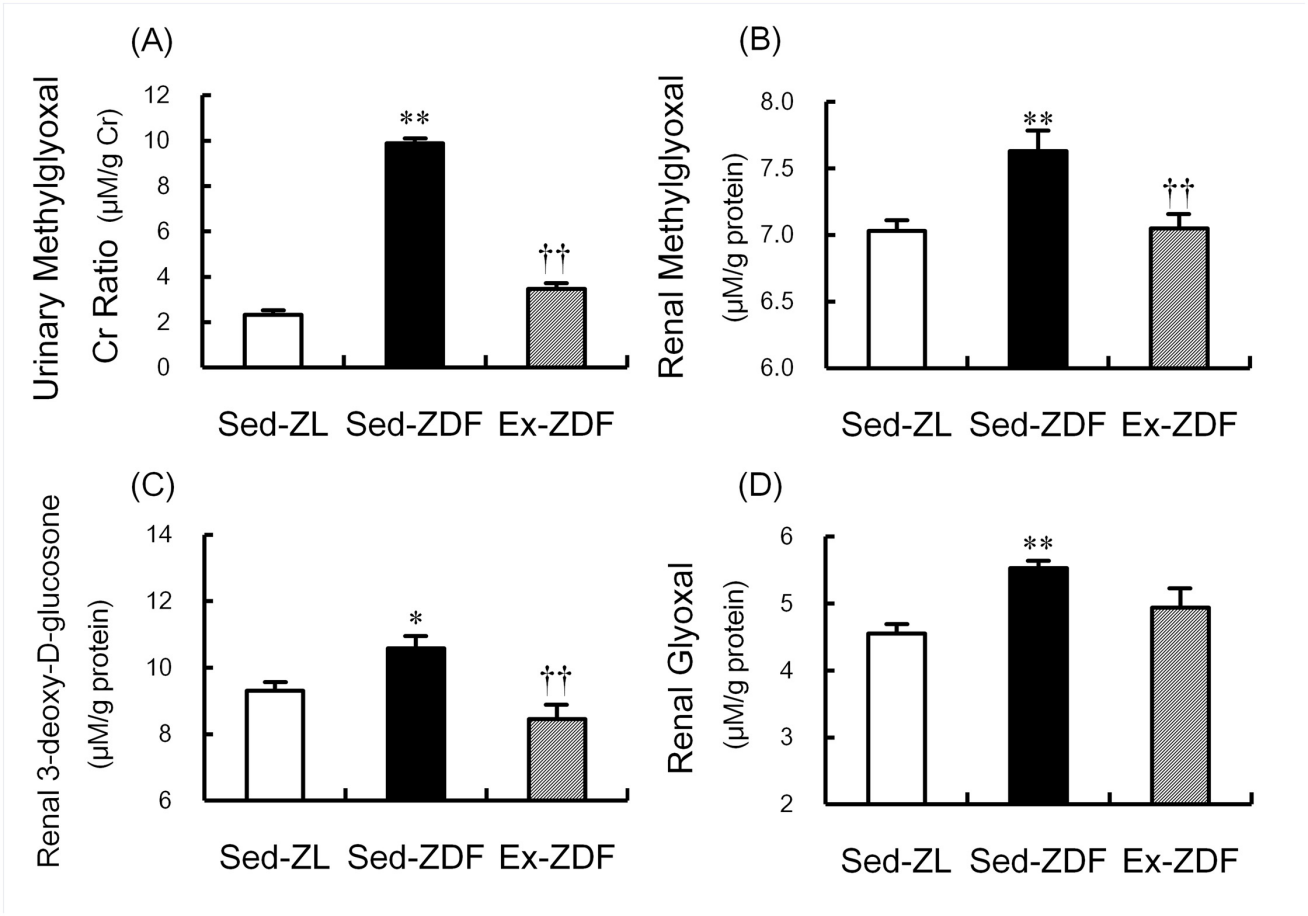
Effects of chronic running exercise on α-oxoaldehydes in Zucker diabetic fatty rats. (A) Urinary methylglyoxal (MG) in sedentary lean Zucker diabetic fatty (ZDF) rats (Sed-ZL), sedentary ZDF rats (Sed-ZDF) and aerobic treadmill exercised ZDF rats (Ex-ZDF) groups was assessed by electrospray ionization (ESI) liquid chromatography (LC)-mass spectrometry (MS) (LC-MS/MS), and expressed as urinary MG:creatinine ratio. (B) MG, (C) 3-deoxy-D-glucosone (3DG) and (D) glyoxal (GO) in the renal cortex of Sed-ZL, Sed-ZDF and Ex-ZDF groups were assessed by LC-MS/MS. Values are means ± SEM (*n* = 5/group). * *p*<0.05, ** *p*<0.01 compared with the Sed-ZL group; ^††^ *p*<0.01 compared with the Sed-ZDF group.

The production of α-oxoaldehydes in the three groups is shown in Fig 8. The urinary methylglyoxal-to-creatinine ratio was approximately fourfold higher in the Sed-ZDF group compared with the Sed-ZL group (*p*<0.01), whereas the ratio was significantly lower in the Ex-ZDF group compared with the Sed-ZDF group (*p*<0.01, Fig 8A). MG, 3DG and GO were significantly higher (by 8.5%; *p*<0.01, 14%; *p*<0.05 and 22%; *p*<0.01, respectively; Fig 8B–8D) in the renal cortex of Sed-ZDF group compared with the Sed-ZL group. Exercise significantly decreased MG and 3DG in the renal cortex of ZDF rats (by 7.6%; *p*<0.01 and 20%; *p*<0.01, respectively; Fig 8B and 8C).

### Glomerulosclerosis, podocyte injury and tubulointerstitial injury

**Fig 9:**
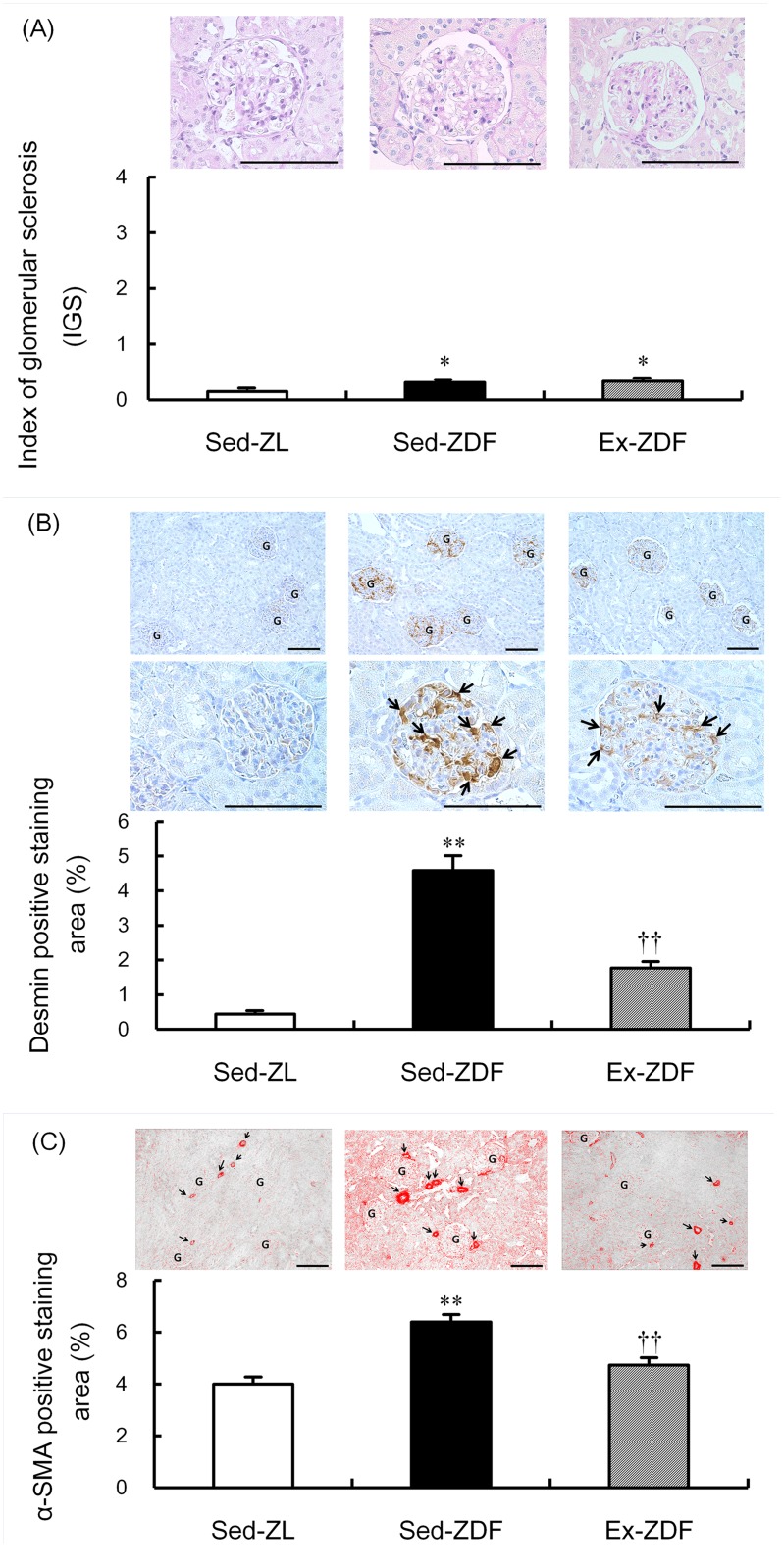
Effects of chronic running exercise on glomerulosclerosis, podocyte injury and tubulointerstitial injury in Zucker diabetic fatty rats. (A) Index of glomerular sclerosis (IGS) was evaluated by Periodic acid-Schiff (PAS) staining in sedentary lean Zucker diabetic fatty (ZDF) rats (Sed-ZL), sedentary ZDF rats (Sed-ZDF) and aerobic treadmill exercised ZDF rats (Ex-ZDF) groups. Upper panels show representative images of renal cortex specimens stained with PAS from the different groups. Original magnification ×200. Scale bar = 100 μm. IGS was determined by grading (0–4) and graphs indicate IGS scores. (B) Podocyte injury was determined by desmin immunostaining of glomeruli in Sed-ZL, Sed-ZDF and Ex-ZDF groups. Top and middle panels show representative images of renal cortex specimens stained with desmin from the different groups. Original magnification ×200 (top), and ×400 (middle). Scale bar = 100 μm. Strongly positive staining regions were observed in glomeruli of the Sed-ZDF group. G indicates a glomerulus in low magnification field (top) and the arrows indicate desmin positive cells such as damaged glomerular podocytes (middle). Graphs depict quantitative representation of desmin positive staining area. (C) Tubulointerstitial injury was determined by α-SMA immunostaining in Sed-ZL, Sed-ZDF and Ex-ZDF groups. Upper panels show representative images of renal cortex specimens stained with α-SMA from the different groups. Original magnification ×200. Scale bar = 100 μm. Strongly positive staining regions were observed in the tubulointerstitium of the Sed-ZDF group. G indicates a glomerulus and the arrows indicate arteries and arterioles with strong positive lesions. Graphs depict quantitative representation of the α-SMA positive staining area. Values are means ± SEM (*n* = 5/group). * *p*<0.05, ** *p*<0.01 compared with the Sed-ZL group; ^††^ *p*<0.01 compared with the Sed-ZDF group.

Scores of IGS (5 grade, 0–4) showed a small significant increase in glomerulosclerosis in both the Sed-ZDF and Ex-ZDF groups (0.31 ± 0.04 and 0.33 ± 0.05, respectively) compared with the Sed-ZL group (0.15 ± 0.02; both *p*<0.05, Fig 9A). Exercise had no effect on IGS scores in ZDF rats. However, the positive staining area of desmin in glomeruli as a marker of podocyte injury was nearly nine-fold higher in the Sed-ZDF group compared with the Sed-ZL group (*p*<0.01), while exercise significantly decreased the degree of desmin staining in ZDF rats by 61% (*p*<0.01, Fig 9B). In addition, the positive staining area of α-SMA, a marker of tubulointerstitial injury, was significantly higher (by 60%) in the Sed-ZDF group compared with the Sed-ZL group (*p*<0.01), while exercise significantly decreased α-SMA in ZDF rats by 26% (*p*<0.01, Fig 9C).

## Discussion

Although exercise has various beneficial effects in T2DM and obesity, the renal effects of exercise and the underlying mechanisms responsible are not yet fully clarified. In the present study, we found that chronic running exercise upregulated renal expression of eNOS and nNOS, and normalized renal NADPH oxidase and α-oxoaldehydes, in ZDF rats at an early stage of diabetic nephropathy. This was accompanied by improvements of urinary albumin excretion, creatinine clearance and renal damage. These data suggest that alterations in eNOS and nNOS expression, NADPH oxidase and α-oxoaldehydes may be potential mechanisms by which exercise improves T2DM and obesity.

It is interesting to note that chronic exercise for 8 weeks resulted in decreased hyperglycemia and hyperlipidemia but with weight gain. Consistent with our findings, treadmill exercise for 8 weeks was previously reported to increase body weight in ZDF rats [29], while chronic aerobic exercise increased total muscle-to-fat ratio without weight loss in obese and T2DM patients [30]. It is possible that exercise may have changed the whole body muscle-to-fat ratio, resulting in increased body weight. Alternatively, the lack of weight gain in Sed-ZDF rats from 12 weeks of age (see Fig 2C) may relate to an insufficient glucose intake and shortage of insulin secretion, which may be restored by exercise (see Fig 3B), presumably due to activation of pancreatic β-cells (see Fig 3E).

Recent studies have demonstrated that pancreatic β-cell failure in addition to insulin resistance are the main pathophysiologic defects in T2DM, and that β-cell failure occurs in early progression in T2DM [1]. It is well established that exercise improves uptake of glucose in muscle, increases insulin sensitivity [1] and insulin release through elevated β-cell function and mass in ZDF rats [6, 7]. From the present results of IPGTT, the insulin levels at all time points in the Sed-ZDF group are insufficient, likely because of β-cell dysfunction. In contrast the insulin levels in the Ex-ZDF group are sufficient, suggesting the maintenance of β-cell function. Indeed, our findings of high levels of HOMA-β in the Ex-ZDF group indicate that exercise may have a protective effect on β-cell function. Consequently (see Fig 3C–3E), the suppression of hyperglycemia is more likely to involve increased insulin secretion by enhanced function and mass of β-cells in ZDF rats, rather than improvement of insulin resistance and sensitivity. The plasma glucose levels in the final samples (Table 1) differ from the levels at baseline in IPGTT (see Fig 3A), and the levels in Sed-ZL of final samples are a little high. As final blood samples were acquired after only 30–60 min fasting, the high glucose value (168 ± 18 mg/dL) in final blood samples of Sed-ZL may be explained by the value at 30 min (186 ± 5 mg/dL) in IPGTT.

In the present study, chronic exercise decreased the NADPH oxidase activity in parallel with p47^phox^ subunit protein expression in the renal cortex of ZDF rats, suggesting that NADPH oxidase through p47^phox^ subunit may contribute to renal O_2_^•–^ in diabetic nephropathy of ZDF rats. Indeed, apocynin, an inhibitor of NADPH oxidase, was recently reported to ameliorate diabetic nephropathy in ZDF rats [31]. Accordingly, the inhibition of NADPH oxidase in the kidneys by exercise in the present study suggests that exercise may alleviate renal oxidative stress, leading to improvement of diabetic nephropathy in ZDF rats.

We previously proposed that exercise has renal protective effects in SHR [19], rats with chronic renal failure [32] and in Goto–Kakizaki rats with nephropathy [33]. In addition, we recently reported that reduced eNOS and nNOS expression in the kidneys is restored by chronic exercise in rats with chronic heart failure [20]. With respect to changes in blood flow, shear stress may be a mechanism underlying the altered expression of renal eNOS in ZDF rats. Increased production of superoxide in the medullary thick ascending limb (TAL) was reported to attenuate NO, leading to reduced medullary blood flow in Dahl salt-sensitive rats [16]. Thus, oxidative stress and NOS in the kidneys may interact in the progression of diabetic nephropathy in ZDF rats, which can be alleviated by exercise to produce renoprotective effects.

In redox signaling, O_2_^•–^ rapidly reacts at a nearly diffusion controlled rate to form ONOO^−^, which substantially limits NO bioavailability [23, 24]. In the present study, nitrotyrosine protein expression (used as an index of ONOO^−^ formation) was significantly lower in the Sed-ZDF group compared with the Sed-ZL group, although there was no significant difference between the Sed-ZL and Ex-ZDF groups, which was contrary to our expectation. One reason for low levels of nitrotyrosine in Sed-ZDF group might be a small amount of NO production. Generated O_2_^•–^ might lead to bioinactivation of NO and cause oxidative damage in Sed-ZDF rats [24].

SBP results showed no hypertension in ZDF rats, in agreement with previous reports using ZDF rats [34] and obese Zucker rats [35], while exercise had no effect on SBP. The higher SBP in the Sed-ZL group may relate to the high activity of ZL rats. It should be emphasized that exercise-induced renal NOS expression may not have a hypotensive effect, but rather an inhibitory effect against renal oxidative stress in ZDF rats that exhibit no hypertension.

It is widely accepted that glomerular hyperfiltration is common in T2DM [36]. We found that creatinine clearance was significantly higher in the Sed-ZDF group compared with that in the Sed-ZL and Ex-ZDF groups, suggesting that exercise may suppress hyperfiltration in ZDF rats. Moreover, glomerular hyperfiltration was proposed to be associated with TGF in ZDF rats [37] and in subjects with T2DM [36]. Thus, the regulation of TGF via increased nNOS in the macula densa of the cortex with exercise may contribute, at least in part, to reduced glomerular hyperfiltration in ZDF rats [10, 17].

In the present study, significant alterations in urinary and renal MG, 3DG and GO suggest that α-oxoaldehydes may play a key role in the progression of nephropathy of ZDF rats. In support of this, chronic exercise was reported to reduce AGEs-specific fluorescence in the renal cortex of Zucker rats [4]. Using accurate and highly reproducible LC-MS/MS quantitation, this is the first evidence that production of α-oxoaldehydes (MG, 3DG and GO) occurs in renal tissues in ZDF and ZL rats, and that MG and 3DG in the renal cortex are normalized in response to exercise in ZDF rats. Under hyperglycemia, AGEs have been reported to induce nephropathy and renal dysfunction, including glomerulopathy, tubulopathy, mesangial expansion and albuminuria [21, 38]. Binding of AGEs to their receptor (receptor for AGE; RAGE) on the tubular surface induces tubular cell injury through NADPH oxidase, which leads to generation of mitochondrial ROS, thereby further increasing oxidative stress in the kidney [21, 22]. These findings suggest that the upregulated interaction of AGEs and NADPH oxidase contributes to the development of diabetic nephropathy. Further investigations are needed to identify the interactions between NOS, oxidative stress, glycation and alternative mechanisms in progression of diabetic nephropathy and effects by aerobic exercise.

Previous studies have investigated the renoprotective effects of aerobic exercise in type 1 diabetic rats [5] and obese Zucker rats [3, 4]. Additionally, a study by our group clarified the renoprotective effects of chronic aerobic exercise in heminephrectomized Goto–Kakizaki rats mainly by evaluation of IGS [33]. To our knowledge, this study is the first to demonstrate the renoprotective effects of exercise by detailed renal histology and to elucidate some molecular mechanisms in a suitable T2DM model. The current study showed that the IGS (PAS staining) in the Sed-ZDF group was slightly higher than that in the Sed-ZL group, while there was no change with exercise. Previous studies evaluating renal damage by PAS staining in ZDF rats have demonstrated no renal injury at an early age (8 weeks of age) [34], but apparent renal damage at approximately 8 months of age [34, 39]. Furthermore, Coimbra et al. [35] reported detailed morphological renal changes in 6–60-week-old obese Zucker rats, with mild renal damage observed from 14 weeks of age, significant focal segmental glomerulosclerosis first noted at 18 weeks and tubulointerstitial damage and proteinuria at 40 weeks. Thus, the intervention phase of the present study (6–14 weeks of age) was in the early phase of diabetic nephropathy progression, prior to the development of severe renal injury. Nevertheless, use of more quantitative immunohistochemical analyses showed that desmin immunostaining was enhanced in the Sed-ZDF group compared with the Sed-ZL group, which was inhibited by exercise, suggesting that exercise is effective in preventing podocyte injury in ZDF rats. Additionally, the mean percentage of α-SMA positive staining area as a marker of arteriole wall thickness was greater in the Sed-ZDF group than the Sed-ZL group, while no differences were observed between the Sed-ZL and Ex-ZDF groups, indicating that renal interstitial injury is also alleviated by exercise in ZDF rats.

Of note, we found no differences in the degree of renal damage between the superficial and juxtamedulla areas of the kidneys using desmin positive staining area assessment. In conditions of hypertension, differing degrees of renal damage between the superficial and juxtamedulla area in the kidneys, which reflect altered response to blood pressure, have been reported [40, 41]. Thus, exercise may have a renal protective effect independent of blood pressure in kidneys of ZDF rats. These data suggest that exercise may be a beneficial therapy, without adverse effects, for patients in the early stage of T2DM without hypertension, rather than the use of antihypertensive medications, including renin-angiotensin-aldosterone system blockers, currently recommended for diabetic nephropathy [42].

In conclusion, upregulated eNOS and nNOS protein and ameliorated NADPH oxidase and α-oxoaldehydes in the kidneys may be potential mechanisms by which chronic running exercise can reduced early diabetic nephropathy in ZDF rats. This phenomenon may not be applicable to all mammals, including humans, and might be influenced by type or status of disease, and dietary effects. However, chronic aerobic exercise does have beneficial effects and may be a novel therapeutic approach for preventing the development of renal dysfunction in patients with T2DM and obesity.
